# Achievement of one health multi-sectoral collaboration in containment of Rift Valley Fever outbreak, Sudan, Red Sea State 2019

**DOI:** 10.1093/eurpub/ckae163

**Published:** 2025-01-13

**Authors:** Hanadi E Hussein, Sara Hassanain, Patrick Okwarah, Hanan Mohamedahmed, Omer Elbadri, Zaafran Alzaki, Mohamed Hashim, Babiker Magboul

**Affiliations:** Federal Ministry of Health, Directorate Health Emergencies and Epidemics Control (HEEC), Khartoum, Sudan; Federal Ministry of Health, Directorate Health Emergencies and Epidemics Control (HEEC), Khartoum, Sudan; Amref International University, Department of Community Health, Kenya; World Health Organization, Department of Health Emergency, Eastern Mediterranean Region, Egypt; Federal Ministry of Animal Resources, Directorate of Disease Control, Khartoum, Sudan; Federal Ministry of Health, Directorate Health Emergencies and Epidemics Control (HEEC), Khartoum, Sudan; Ministry of Health, Red Sea State, Sudan; Ministry of Animal Resources, Red Sea State, Sudan; Federal Ministry of Health, Directorate Health Emergencies and Epidemics Control (HEEC), Khartoum, Sudan

## Abstract

Rift Valley Fever is endemic in Sudan, with a notable outbreak declared in 2019, affecting multiple states. In this study, we examine the Red Sea State, Sudan’s experience in applying the One Health approach, to contain Red-Sea RVF outbreak. A retrospective analysis of national and sub-national data and a review of literature were conducted to assess the application of One Health response and to derive lessons learned. The analysis revealed a total of 576 human cases and two deaths, with a case fatality rate of 0.35%, from 25 September 2019 to 25 January 2020. Most cases (99%) were from the Red Sea and River Nile States, and only six sporadic cases were from other five states. The Red Sea State reported 322 human and 74 animal cases, including 74 abortions and 12 animal deaths. Triggers and risk factors include floods, uncontrolled movement of animal, close contact with animals, poor disposal, and unsafe burial practices for animals. One Health approach was utilized all through the defeat of outbreak. A multi-sectoral response plan was implemented, leading to the declaration of the end of the outbreak in 2020 which was reviewed and lessons were derived. One Health approach provided a coordinated action between health, veterinary, and environmental authorities at national and subnational levels. Synergistic efforts have minimized risk of RVF spreading among human and animal. The experience was leveraged to strengthen response approaches for zoonotic diseases. Structural and capacity gaps and financial constraints were identified as implementation challenges.

## Introduction

Rift Valley Fever (RVF) is a viral illness that affects humans and animals. It can be transmitted to humans through exposure to infected animals, either directly through their blood, body fluids, or tissues or indirectly through mosquito bites [1]. RVF is able to infect many species of animals causing severe disease in domesticated animals including cattle, sheep, camels, and goats. Sheep and goats appear to be more susceptible than cattle [2]. RVF can exhibit a range of symptoms, such as abortion and fever in animals, flu-like symptoms and hemorrhage in humans, and can be fatal for both [2]. In sheep, abortion rates can be as high as 100%, particularly in young and foreign breeds, and adult animals may experience mortality rates ranging from 5% to 60% [3].

Numerous countries in sub-Saharan and East Africa have experienced RVF epidemics and the disease has recently spread to new regions [4]. It started in the Great Rift Valley since 1930 and most major epidemics have been reported in Kenya (1997–1998, 2006–2007), Tanzania (2007), Somalia (2007), and Mauritania (2010, 2012) [5], while Sudan was severely affected by the large RVF outbreaks that occurred in East Africa during 2007 and 2010. These recent outbreaks were more severe among associated human cases, which could be a reflection of suboptimal case picking, case finding, diagnosis, and surveillance during earlier outbreaks [6].

RVF can have a significant impact on affected communities and can result in substantial economic losses due to livestock deaths and trade or export bans imposed [4, 7]. In humans, RVF typically results in severe complications or death in 1%–5% of cases [8]. Moreover, outbreaks often occur with flooding, which can lead to the emergence of infected vectors, causing extra burden while putting communities at greater risk and vulnerability during the disaster [9].

Sudan, a country endemic for RVF, has considerable environmental risks, including heavy rainfall and flooding. Expansion of irrigated agricultural schemes and the construction of dams in Northern region have introduced the RVF to new areas of Sudan. Furthermore, Sudan has a large number of mosquito species that transmit RVF. Given its large livestock population, vulnerable groups to RVF; such as veterinarians, farmers, butchers, animal handlers, and nomads, are numerous. Sudan has experienced notable outbreaks of RVF and as East African recent outbreaks, they were associated with more human morbidity and mortality [6]. In its 2007 outbreak, there were 747 confirmed human cases and 230 fatalities, resulting in a case fatality rate of 30.8%. The outbreak was impactful, with high mortality and abortion rates among infected animals [10]. The subsequent outbreak occurred in 2019, primarily affecting the Red Sea and River NileStates both animal and human.

Ultimately, the prevention and control of RVF require a multifaceted approach that includes enhancing public system resilience [7]. One Health is a comprehensive, unified strategy to balance and improve health of people, animals, plants, and ecosystems in a sustainable manner [11]. It acknowledges interdependence and relationship between human health, domestic and wild animals, plants, and the larger environment, including ecosystems [11]. It mobilizes various sectors, disciplines, and communities at different levels to promote well-being and address threats to health and ecosystems. It also addresses shared needs for clean water, energy, air, safe and nourishing food, action on climate change, and sustainable development [12].

Sudan adopted the “One Health” concept to address threats to humans, animals, and environment, as a platform for multisectoral actions. This focus intensified after the Joint External Evaluation (JEE) in 2016 recommendation of strengthening joint approaches [13]. The Federal Ministry of Health (FMOH) and the Federal Ministry of Animal Resources (FMOAR), as supreme governance bodies have implemented “One Health approach” and utilized it to manage 2019 RVF outbreak through governance, coordination, field operations, monitoring, and oversight at national and sub-national levels. This paper aims to describe the Red Sea State, Sudan's experience in applying the One Health to contain the outbreak.

## Methods

### Study design

The study adopted a descriptive case-study design. It involved a description of routine surveillance data and reporting of a desk review for steps taken in alignment to One Health approach at national and sub-national levels in containing the RVF outbreak in Red Sea State in Sudan in 2019. It utilized the verified and approved surveillance data and reviewed grey documents approved by the Federal Ministry of Health (FMOH) concerning the 2019 RVF outbreak.

### General setting

Sudan is a country located in Africa with an estimated population of 42 million people and 140 million livestock, comprising mainly cattle, sheep, goats, and camels in 2009 [14]. It has a diverse ecology of deserts, semi-deserts, and low- and high-rainfall zones. There are approximately six major dams in different parts of the country.

### Specific setting

The Red Sea State is one of the 18 states in Sudan and situated in eastern part. It is characterized by a desert climate with sweltering summers and moderately hot winters and a coastal-marine ecosystem where rainfall occurs during the four months of winter. Spanning an area of 212 800 square kilometers, the state shares its northern border with Egypt and its western and southern boundaries with the River Nile and Kassala states, respectively. It is administratively divided into ten localities and has the Arbaat Dam as a water source. The livestock estimate in the Red Sea state is 1 620 677 heads of livestock with nomadic free movement across localities and bordering states [15]. The state experienced an outbreak of RVF in 2019, which affected both humans and animals, and has also recorded outbreaks of mosquito-borne diseases, including Dengue Fever [16] and chikungunya [17], in recent years.

### Data collection and analysis

#### Data source

The quantitative part utilized routine secondary data collected and reported retrospectively during the RVF outbreak, which was classified under List A of the Notifiable Diseases of the National Surveillance System, from September 2019 to February 2020. The qualitative data were from approved grey reports and blueprint documents generated for the RVF, 2019 outbreak containment in Sudan.

### Data collection and quality management

Data were extracted from surveillance system using a customized Excel sheet, encompassing suspected and confirmed cases reported in the Red Sea State from 19 September 2019 to 17 February 2020 following a sorted permission. The unidentified data were verified and triangulated at both sub-national and national levels using outbreak notifications, weekly epidemiological reports, mission reports, action reviews, plans, and coordination meetings’ minutes.

A tool encompassing the One Health approach components was deployed to gather data from grey materials on the RVF-Red Sea State outbreak and related documents. The review included documents related to outbreak response thematic areas (Coordination, Surveillance, Case management, Risk communication and social mobilization, Laboratory and integrated vector management control, and Infection prevention control), including weekly, monthly, and final/after-action reviews; outbreak reports; coordination meeting minutes and recommendations; official notifications; and declarations from the two ministries. Irrelevant or non-approved documents were excluded. The research team meticulously reviewed, verified, organized, and interpreted the data to report on the containment of the Red Sea outbreak and the lessons learned in alignment with the One Health approach implementation. Additionally, a basic literature search was conducted using Google Scholar on One Health and RVF, complemented by targeted searches on specialist websites such as WHO and WOAH.

#### Data analysis

This case study involved a descriptive quantitative analysis of retrospective secondary data extracted from already published surveillance data. The number of reported cases was calculated in a series of times, and the pattern was plotted on an epidemiological curve that described the onset and end of the outbreak. Case fatality rates were also calculated. A qualitative thematic content analysis was used to explore the context and understand the whole response as well as the perceived lessons under the onehealth approach.

## Results

### Magnitude of the multi-state outbreak

**Figure 1.**
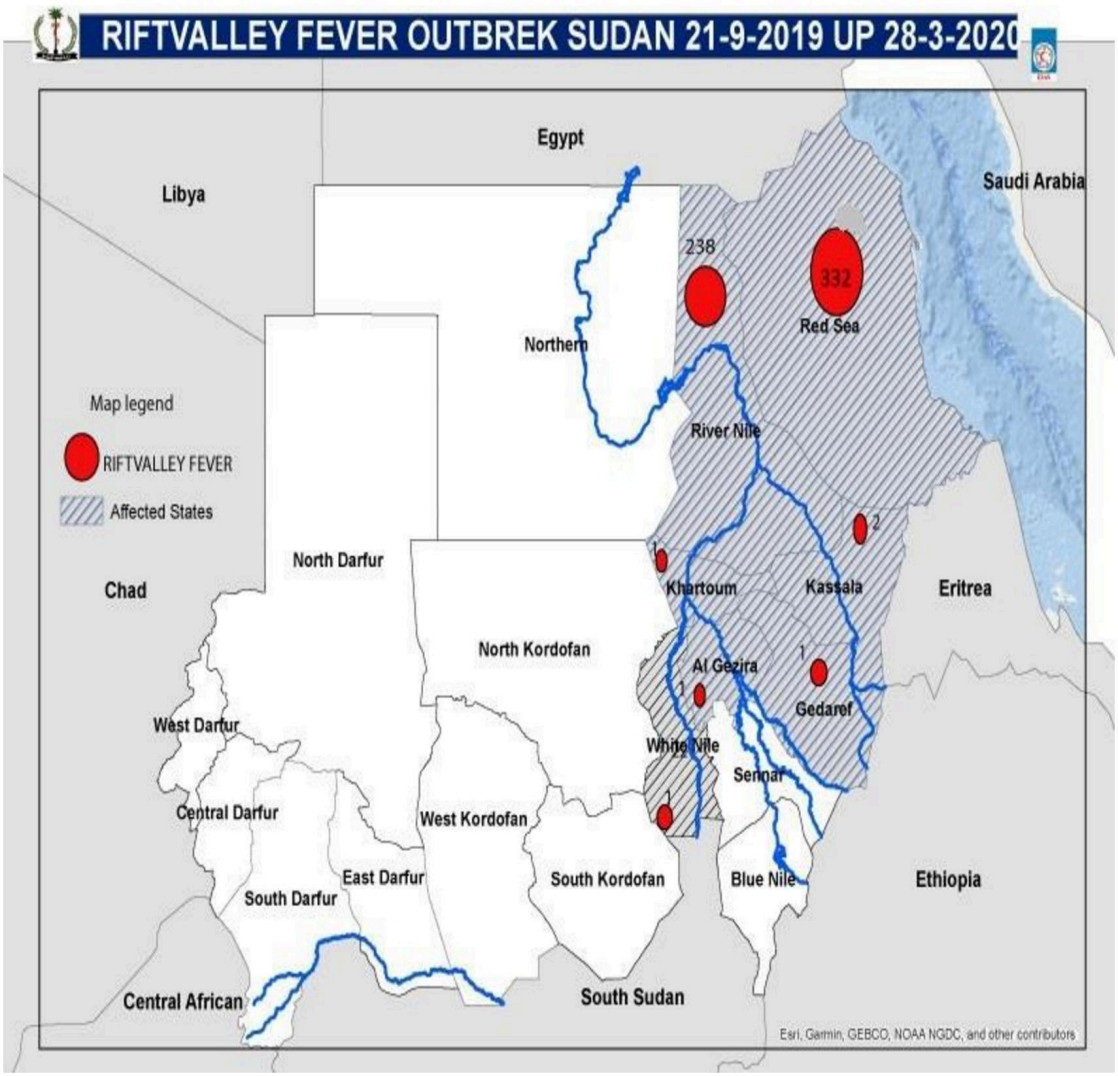
Map for human cases of Rift Valley Fever in Sudan during the outbreak of 2019–2020.

**Figure 2.**
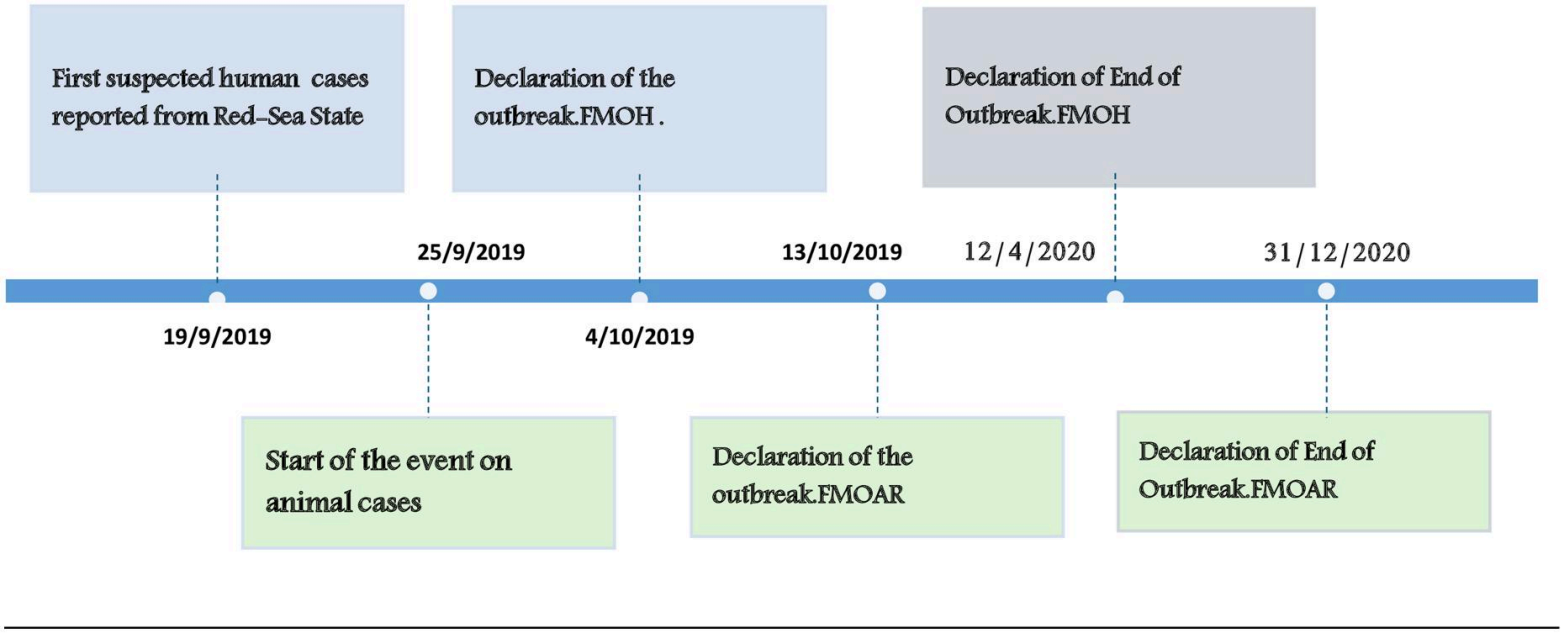
The milestones of the Rift Valley Fever outbreak in Sudan 2019–2020.

**Figure 3.**
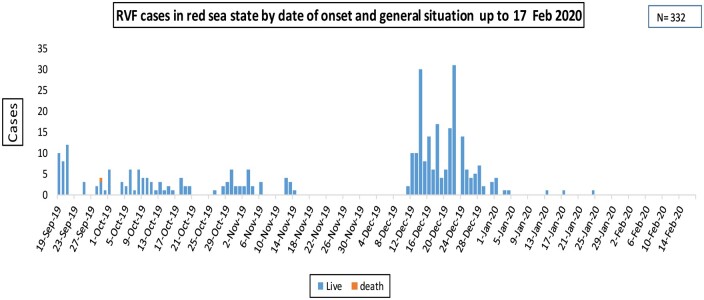
The epidemiological curve of Rift Valley Fever cases from the date of outbreak onset until 17 February 2020.

The RVF outbreak in 2019 was a multi-state outbreak that mainly affected Red Sea and River Nile States (Fig. 1). Six sporadic human cases were confirmed in more than five states; however, field investigations by the Ministry of Animal Resources did not report any animal cases in these foci of the five states (Fig. 1). Beginning on 19 September 2019, and lasting through 25 January 2020, the outbreak resulted in 576 human cases, with two fatalities (CFR = 0.35%) (Figs 2 and 3). Among the 160 samples tested, 67 (41.9%) were found to be positive for RVF by Polymerase Chain Reaction (PCR) (Fig. 3). This test was conducted at the National Public Health Laboratory (NPHL) [18]. Also 74 cases of abortions and 12 deaths in animals associated with RVF in humans were reported [19].

### Magnitude of the Red Sea outbreak

Of the overall outbreaks, the Red Sea state constituted 332 cases of RVF and two deaths (Fig. 3). The cases were from the Toatlal and Tohashban villages in the Arbaat, Algonob, and Aloleb localities. The two villages are very close to the Arbaat Dam. Human cases presented with high fever, headache, and joint pain, and two deaths were reported to FMOH. Abortion has been reported among animals. The number of human cases showed a significant decrease until November 2019. Another wave of cases was reported on 12th of December from Hosheri, Port Sudan. A total of 21 abortions and 4 deaths among goats in Red Sea State, Elgounob, and Aloleb localities were found [18], and the outbreak was confirmed in goats through Enzyme-linked Immunosorbent Assay (ELISA) [19].

### Steps taken for utilizing the one-health approach

The One Health application tool was adopted in response to the guidelines of the (JEE) of IHR (2005) which was used in Sudan in 2016 to evaluate Sudan scale on the 19 IHR core capacities, including zoonotic diseases. JEE defined strengths, challenges, and recommendations for priority actions. Also, the One Health Zoonotic Diseases Prioritization tool (OHZDP) CDC tool was used to grasp recommendations for multisectoral action plans. The One Health was applied through the containment pillars and the following steps were taken.

#### Compliance with international organization regulations

The steps included ensuring compliance with the International Health Regulations (IHR) (2005) [20] and the World Organization of Animal Health (WOAH) regulations [21]. Following IHR 2005, National Focal Point reported confirmed RVF cases to the Eastern Mediterranean Regional Office (EMRO)—World Health Organization (WHO) through the WHO Sudan Country Office [18]. Federal (MOAR) has reported a confirmed outbreak in goats and sheep [19].

#### Coordination of preparedness and action

An early setup of the national Emergency Operations Centre (EOC) using an Incident Management System (IMS) for quick effective coordination was in place. A national, joint RVF commitess and technical working groups were timely established involving decision-makers (ministers, undersecretaries of Health and Animal Resources, directors at federal and state levels of FMOH and FMOAR), community representatives (local leaders, volunteers from resistance committees, and affected localities), international organizations [WHO, WAHO, the Food and Agriculture Organization (FAO)], professionals, and other partners (ministries of Finance, Trade, Federal Governance, Agriculture and Irrigation, Meteorological Authority, and local Security and Police forces) and they identified context-specific risk factors and governed the coordination. Although the multi-hazard preparedness and response plans for 2019, including vector-borne diseases, were in place yet, most high-risk states had partially implemented them [18]. Hence the plans were updated accordingly, national RVF guidelines were established, and supplies and other medical consumables were prepositioned by national teams in the states as part of the flood preparedness actions. A functional Rapid Response Team (RRT) was deployed at both national and state levels.

#### Surveillance

The surveillance system was functioning at the state and community levels, and the community-based surveillance, implemented in 2017, proved to be resourceful. Trained public health and veterinary surveillance staff at both the national and state levels were deployed. Case definitions, reporting tools, line lists, and case investigation forms were prepared prior to the response. Data flow steps and timelines of reporting and methods for reporting and analysis at different administrative levels were clearly explained in the surveillance guidelines. Zero reports were activated at all states since epidemiological week 36, 2019. All reporting health facilities, in addition to 1755 selected sentinel sites in week 38, 2019, used to share their daily reports [22]. The Sensitivity of surveillance in searching for suspected cases was increased, where 88.4% of reports were received within 24 h from the lower levels to the national level [22]. The case-based surveillance data were eventually shared in a timely manner.

#### Laboratory diagnosis capacities

Diagnostic facilities were available at the NPHL and the Central Veterinary Research Laboratory (CVRL). At NPHL, initial and follow-up screening was conducted using Reverse Transcription Polymerase Chain Reaction (RT-PCR) with ELISA screening, while RT-PCR was not routinely used for RVF diagnosis at CVRL. Coordination focal points between NPHL and CVRL are imposed, facilitating the exchange of training activities for RVF serology diagnosis, while transparent reporting of cases and progress were maintained for both ministries.

#### Response

Medicines, medical consumables, and laboratory supplies were positioned at facilities by FMOH as part of rainy season preparedness. Designated treatment centers for RVF were identified in the affected localities, and Hosheri was rehabilitated by the WHO to manage suspected febrile patients [23].

#### Distribution of updated RVF case management guidelines

Joint outbreak investigation and response teams were deployed at federal, state, and locality levels from the MOH and MOAR, working in collaboration to control the outbreak at the human-animal interface [18]. Expert epidemiologists from each side exchanged knowledge and experience, and the WHO and FAO provided government sectors with technical and logistical support. Vector control capacities and resources, such as the National Integrated vector management strategy, the staff trained on entomology, and vector control activities, were utilized. The active engagement of local communities in vector control activities such as disposal of water-holding containers reduced the rates of indoor breeding.

Ten days of a joint awareness campaign consisting of a community-based surveillance team (health promoters) and communication officers (veterinarians) was conducted in Algonob and Aloleb localities. Additionally, federal support with vector control tools and equipment, supervisory missions to identify vulnerable locations, and integrated vector surveillance helped in minimizing the transmission of RVF. The enforcement of the Epidemic Diseases of Animal Act (2001), in coordination with the police force, has also restricted animal movements [23] Veterinary checkpoints were established to control the spread of the disease in line with the 2001 Act [24]. Veterinary authorities, implemented preventive measures, such as proper disposal of aborted fetus and dead animals and disinfection of houses and animal sites. A veterinarian was stationed at the health facility to receive reports, investigate rumors of suspected animal cases, and deliver health education messages to livestock owners. The end of this outbreak was declared in April 2020 [25]. An after-action review process was conducted in September 2020 [26].

## Discussion

The outbreak transpired over roughly five months, exhibiting a low case fatality rate. Its activation may be linked to the Red Sea coastal area, irrigated agricultural ecology, and rainy seasons, where close animal contact significantly influenced transmission. A 2007 study on Saudi and Sudan outbreaks highlighted how climate and ecological factors are associated with RVF spread [27]. The Red Sea outbreak confirmed animal-to-human infectivity, and this documentation and other studies validated the One Health approach's effectiveness in addressing RVF outbreaks and in identifying wider range of affected stakeholders who needs to act in an integrated manner [28]. This coordinated partnership between communities, decision makers, development partnets among others, exemplified the One Health approach, and enabled the joint rapid response team to identify context -specific risk factors. It also provided a practical exercise of the One Health approach in mediating animal-human- ecosystem’ investigations by joint team through addressing integrated vector management and risk communication related to social habits and practices, particularly those involving animals, their resources, morbidity, and mortality. This case study demonstrates that the one health approach and its application was guided by the identified gaps, utilizing the JEE-IHR (2005) and CDC-OHZDP tools, the implementation faced facilitating factors and barriers and generated certain recommendations which then informed multisectoral engagement plans (Supplementary Box). By implementing the One Health approach throughout the RVF outbreak response, containment efforts were enhanced through improved synergy and coordination between the Health and Animal Resources ministries at both national and local levels. This bottom-up strategy boosted efficiency and tackled financial shortfalls, which significantly enhanced the response. A research has once demonstrated viability of the one health application in public health issues and specifically its financial efficiency in defating RVF [29].

Sudan experience involved local communities and at risk-populations in the Red Sea State, including herdsmen and animal owners. The literature emphasizes that such engagement is vital for bolstering prevention, early detection of animal and human cases, and mounting an effective response [30].

One Health coordination also facilitated the collection of animal and health data, crucial for preventing delays as seen in the 1997 Kenya epidemic, where numerous human deaths occurred before RVF was identified [27].

Vector management was integral to the One Health response, in fostering collaborative discussions and comprehensive entomological insights in the State. Enhancing vector control collaboratively is a cost-effective strategy with potential benefits for countries with multiple vectors, like Sudan [27]. Systems thinking increased overall RVF awareness and improved problem-solving while avoiding siloed planning, enhancing governance and programmatic responses to zoonotic diseases. For example, the establishment of supreme and technical committees for ministers and undersecretaries promoted high-level political will, monitoring, and oversight. The timely initiation of multi-sectoral EOC provided a platform for integrated, prompt identification of micro-challenges and solutions. To tackle the issue of centralized and slow diagnosis at FMOH and FMOAR, which previously impeded the uptake of results from distant locations, several effective measures were implemented. These included establishing focal points and a collaborative committee to facilitate coordination between NPHL and CVRL, implementing uniform laboratory request forms and protocols, and shortening the time required for result delivery. Joint investigation teams and designated treatment centers with veterinarians and updated case management guidelines supported timely detection, diagnosis, and management, and enhanced reporting and risk communication as critical interventions [31]. The approach also helped in joint desk and dossier development for after-action review, leading to the declaration of the end of the outbreak and the revival of livestock movement and investment. The consultative After-Action Review (AAR) process as a linked evaluation tool, had helped further in verification of facilitating factors and barriers, and in recommending the way forward to sustain the one health approach (Supplementary Box) [26].

Overall, lessons learned were that “One health” adoption enhances outbreak response by promoting more holistic, proactive, and integrated strategy that addresses complex factors influencing disease spread. Also, developing a structured tools is crucial for streamlining outbreak management and response to zoonotic diseases which have been also emphasized after 2019, by the tripartite tools of WHO, FAO, and WOAH. Leveraging “One Health” experience and AAR recommendations, on August 2021, a One Health Zoonotic Diseases Prioritization (OHZDP) workshop for multisectoral engagement was held and identified RVF as Sudan's top priority zoonotic diseases [32]. Subsequently, a Joint Risk Assessment conducted on August 2021, indicated the RVF's high likelihood and moderate impact [33]. This “One Health approach” can be sustained and continuously adapted to meet public health challenges through maintaining long-term integrated and effective collaboration across the human, animal, and environmental sectors. Policy integration and institutionalization, emphasizing roles and responsibilities through measurable outcomes, mobilization of resources and funding, capacity building, public awareness, and community engagement are all central in sustaining one health approach.

## Conclusion

An added value of applying a One Health approach to control the RVF outbreak in Red Sea State in 2019 was the containment of the outbreak and the restoration of livestock export operations in a short period of time in comparison to previous outbreaks in Sudan. It also institutionalized the road map for implementing One Health concept in strategic planning to control other zoonotic diseases. The practical utilization of One Health is highly recommended. Strengthening of coordination mechanisms and establishing of the One Health platform could facilitate the implementation of integrated activities to prevent the occurrence and transmission of zoonotic diseases and improve human and animal health. Control of RVF outbreaks requires collaboration among medical and veterinary clinicians, public health specialists, epidemiologists, and laboratory technologists to perform preventive measures, rapid investigations, and treatment of the affected patients and animals. Multidisciplinary studies are needed to address the whole RVF cycle and to strengthen the evidence-based prevention and control at the contexualized level.

## Supplementary Material

ckae163_Supplementary_Data

## Data Availability

All data is presented in the manuscript and it is from surveillance system held by the federal Ministry of Health, which is not publicly accessible as part of the ministry of health surveillance data management standards. Additional approved grey literature materials can be found in Federal Ministry of Health's official website, (https://fmoh.gov.sd/fmoh_new/) and the repository of the health emergency and epidemic control directorate of the Federal Ministry of Health. Key points The RVF outbreak in 2019 was a multi-state outbreak that mainly affected the Red Sea and River Nile States. Cases of abortions and deaths in animals associated with RVF were reported. The utilization of “One Health” approach in the Red Sea State Outbreak has synergized efforts and increased harmonization and synergy between FMOH, FMOAR, WHO, FAO, and other partner hence effective outbreak containment. The operationalization of the One Health concept at the national, state, and locality levels is highly recommended to impose joint capacity building to control RVF. Multidisciplinary research is crucial for addressing the entire RVF cycle and strengthening evidence-based prevention and control.
