# Supplementary material for: Achievement of one health multi-sectoral collaboration in containment of Rift Valley Fever outbreak, Sudan, Red Sea State 2019
Source: Eur J Public Health. 2025 Jan 13;35(Suppl 1):i66–72. doi: 10.1093/eurpub/ckae163 (PMC11725951; doi:10.1093/eurpub/ckae163)
Supplement: ckae163_Supplementary_Data [file ckae163_supplementary_data.pdf]

Facilitators:

1. Timely activation of the EOC resulted in effective coordination, resource mobilization, public awareness through the instrument of EOC via Press briefing, radio, and TV.
2. A transparent approach was followed in the official notification and declaration of the event, enabling initiation of early response, support from stakeholders, enhanced public awareness, joint investigation, and response between MOAR and FMOH.
3. Effective coordination mechanisms were in place that helped sharing of surveillance data and joint implementation of response activities.
4. Good coordination with related sectors and partners at the state level after the notification of the outbreak.
5. Regular frequent meetings of the technical task force were held within short intervals at federal and state levels.
6. FMOH and Red Sea State multi-sectoral task force developed RVF One health response plan jointly with MOAR.
7. Meetings of Ministers, Undersecretaries, Directorate General and the sectors committees of the Council of Ministers facilitated decision-making and distribution of roles and responsibilities.
8. Undersecretaries from FMOH and FMOAR deployed a joint mission led by epidemiologists to provide technical support to the affected state. The federal team accompanied by public health specialists from WHO country and sub offices visited the affected localities and supervised the development of a One Health response plan.
9. Effective coordination in risk communication activities, media engagement, deployment of Information Education Communication (ICE) materials were also good practices identified during the outbreak. Community participation and coordination raised the alert, and the direct communication (home visit and group discussion) raised the awareness on how to protect the community by adopting healthy behaviours.
10. A wide range of stakeholders was involved in RVF outbreak response.

Barriers:

1. Insufficient human resources namely risk communication officers to maximize carrying out risk communication activities as planned, and low community participation due to religious and cultural beliefs.
2. Lack of sufficient funding to the response from government, United Nations organizations and other donors delayed the implementation of response activities.
3. Countries banned the importation of livestock from Sudan with negative impact on national economy.

Recommendations:

1. Leveraging the lessons learned is also essential for further prevention and control. Thus, mobilization of resources and capacity building of Joint Rapid Response Teams at state and locality levels, integrated vector management, integrated surveillance, and expansion of community - based surveillance system between MOH and MOAR is prioritized.
2. Increasing the awareness among animal owners towards RVF through a budgeted and integrated risk communication plan to ensure timely reporting and engagement in prevention and control of disease Ensuring availability of the health and veterinary services at the localities and improving capacities at local level is crucial. Moreover, institutionalization of coordination between CVRL & NPHL including exchange of experience and resources and sharing of data is essential. The enforcement of the legislation that monitor the livestock movement during outbreaks and adopting compensation policies for animal owners is also needed.

Supplementary Box: The findings on the RVF Outbreak Response. (26)
